# A Bead-Based Nonradioactive Immunoassay for Autoantibody Testing in a Mouse Model of Myasthenia Gravis

**DOI:** 10.3390/antib13030053

**Published:** 2024-07-01

**Authors:** Afrin Bahauddin, Kyra Curtis, Jutatip Guptarak, Ruksana Huda

**Affiliations:** Department of Microbiology and Immunology, University of Texas Medical Branch, Galveston, TX 77555, USA

**Keywords:** acetylcholine receptor, anti-acetylcholine receptor antibody, myasthenia gravis, autoantibody, autoimmunity

## Abstract

Serological testing for anti-acetylcholine receptor (AChR) autoantibodies is not only crucial for the diagnosing, disease monitoring, and treatment management of patients with myasthenia gravis (MG) but also for preclinical studies utilizing MG disease models. However, there are no specific guidelines on which methods to use in clinical diagnostic or research laboratories to detect or quantify any MG-specific autoantibodies. Conventional autoantibody assays, particularly those for anti-AChR antibodies, are varied and mostly laboratory-specific. Here, we report our new nonradioactive immunoprecipitation–immunoblotting method for assessing autoantibodies (anti-AChR antibodies) in a mouse model of MG. This simple, efficient, reproducible, and cost-effective assay appears superior to the enzyme-linked immunosorbent assay but comparable to the radioimmunoprecipitation or cell-based assay in specificity and sensitivity. Thus, the newly developed assay can serve as a valuable alternative to classical assays and is suitable for routine testing of AChR-specific autoantibodies in preclinical studies. The further optimization of our assay may facilitate its application in the diagnosis and therapeutic management of patients with MG.

## 1. Introduction

Myasthenia gravis (MG) is a chronic debilitating autoimmune disorder characterized by the dysfunction of the neuromuscular junction (NMJ). MG leads to muscle weakness, primarily due to the autoantibody-mediated scarcity of postsynaptic functional acetylcholine receptors (AChRs) at the NMJ [1]. Anti-AChR autoantibodies can deplete muscle AChRs through three known mechanisms [2]. The first mechanism involves the anti-AChR autoantibody-mediated binding of AChRs, resulting in complement activation, followed by membrane attack complex formation, cell lysis, and, thus, AChR depletion from the muscle endplate (a common mechanism underlying MG). The second mechanism involves the anti-AChR autoantibody-mediated modulation (cross-linking) of AChRs, resulting in their internalization and degradation, causing the reduced surface availability of functional AChRs. The third mechanism—the less common one—involves the anti-AChR autoantibody-mediated blocking of AChRs, preventing the receptor binding of acetylcholine neurotransmitter. A common theme across the three mechanisms is that the loss of functional AChRs leads to impaired neuromuscular signal transmission and resultant muscle fatigue. Thus, clinical signs of MG typically include ptosis, diplopia, slurred speech, appendage tremors, and even respiratory failure due to worsening conditions [3]. Thus far, more than 700,000 cases of MG—either generalized MG (affects body muscles) or ocular MG (affects eye muscles)—have been reported worldwide [4]. A mouse model of MG (experimental autoimmune MG [EAMG]), established through the direct immunization of mice with Torpedo AChR, has been demonstrated to effectively reflect the major features of generalized MG [5].

Nicotinic AChR comprises five subunits: α, β, δ, adult-type AChR-ε, or adult-type AChR-γ subunits [6]. Approximately 85% of patients with generalized MG and 50% of those with ocular MG show the presence of AChR-specific antibodies against all five subunits of nicotinic AChR [7]. Furthermore, 5–10% of patients with MG exhibit anti-muscle-specific tyrosine kinase antibodies, regardless of their AChR serological status (seropositive or seronegative). Current serological assays for testing autoantibodies can detect either AChR or muscle-specific tyrosine kinase-type autoantibodies. The pathogenic subclasses of AChR autoantibodies in human and mouse models are IgG1 and IgG2b, respectively, and IgG4 and low levels of IgG1–IgG3 are the MuSK antibodies for both mice and humans. Although 1–15% of all patients with MG are found to be seronegative in conventional or classical assays [8], e.g., the enzyme-linked immunosorbent assay (ELISA), the improved radioimmunoprecipitation assay (RIPA) and cell-based assay (CBA) have proven effective in detecting autoantibodies in some of these patients. Moreover, these assays can detect MG-specific autoantibodies against certain NMJ-associated antigens, including low-density lipoprotein receptor-related protein 4 (in approximately 2% of all patients with MG), titin, ryanodine receptor, collagen Q, and agrin (infrequent detection) [8]. However, each of these assays has some limitations.

ELISA is the method of choice for routine antibody testing in academic research laboratories with limited resources and many diagnostic laboratories in the industry. Although ELISA is a sensitive method, it can sometimes lack specificity because of high background noise levels or false-positive results stemming from nonspecific binding due to cross-reactivity. In addition, sample contamination during the process and colorimetry-based end-point measurement can compromise precision, accuracy, and, thus, data reliability.

RIPA or radioimmunoassay is considered to be the gold standard method for measuring anti-AChR antibodies in serum or AChR levels in muscle samples [9]. Despite its simplicity, relatively few steps, and high sensitivity and specificity in target detection, RIPA is a cumbersome process because of the need for a radioactive agent use permit, as well as specialized facilities, equipment, and skills. Moreover, the use of radioisotopes carries health and environmental risks. As an alternative to RIPA, fluorescence-labeled AChR assays have been developed. However, these assays are less sensitive than RIPA and require specialized equipment. In live or fixed *CBA*, transiently or stably expressed clustered AChRs in cell lines capture anti-AChR antibodies in serum. Although this method outperforms ELISA and RIPA in terms of sensitivity and specificity, the use of CBA is limited because of its complexity, the need for a highly specialized fluorescence-activated cell sorter, skilled operators and analysts, and challenging reproducibility. The *dot blot* analysis of anti-AChR antibodies exhibits a sensitivity similar to that of RIPA and involves the use of AChR antigens immobilized onto a nitrocellulose membrane [10]. However, this method produces a high background noise on the blot when testing whole sera; therefore, optimal dilutions are essential for dot blotting.

Herein, we describe an alternative and efficient approach for detecting and quantifying anti-AChR antibodies. Our method combines immunoprecipitation and immunoblotting assays involving biotin and streptavidin.

## 2. Materials and Methods

### 2.1. Mouse Model of MG

Male C57BL/6 mice (age: 8 weeks; weight: ~20 g) were purchased from Jackson Lab (Bar Harbor, ME, USA) and housed in a barrier facility at the University of Texas Medical Branch, United States. The mice were maintained following the guidelines issued by the National Institutes of Health and the University of Texas Medical Branch Animal Care and Use Committee.

To induce MG disease in mice, affinity-purified Torpedo AChR (in-house purified or purchased from Medix Biochemica, FL, USA) @ 20 μg/mouse was emulsified in complete Freund’s adjuvant or CFA (BD Difco, NJ, USA) with phosphate-buffered saline (PBS). Details on the EAMG induction are in our previous article [11]. In brief, emulsions of CFA with PBS alone or CFA with AChR and PBS (the immunogen) were subcutaneously injected into the hind footpads and shoulders of the mice. After 4 weeks, two booster immunizations were performed on the shoulders and thighs of the mice. MG disease in the mice manifested by measurable clinical signs of muscle weakness [11]. Blood samples were collected from the EAMG mice through tail vein bleeding for the testing of serum anti-AChR autoantibodies.

### 2.2. Detection of Pathogenic and Anti-AChR Autoantibodies

Pathogenic anti-AChR immunoglobulin (IgG/IgG2b) levels in EAMG mice were measured using our newly developed nonradioactive, two-step immunoprecipitation–immunoblotting assay. This assay relies on the unique affinity of biotinylated α-bungarotoxin (BTX) to AChR (added to serum samples) and subsequent binding of the AChR to AChR-specific autoantibody present in the plasma or serum to form biotinylated BTX–AChR-anti-AChR Ab complex. Upon the addition of streptavidin magnetic beads, the complex tightly binds to and immobilizes these beads. Next, a magnetic field is created to separate the beads, and purified antibody complexes are eluted from the streptavidin beads.

**Figure 1:**
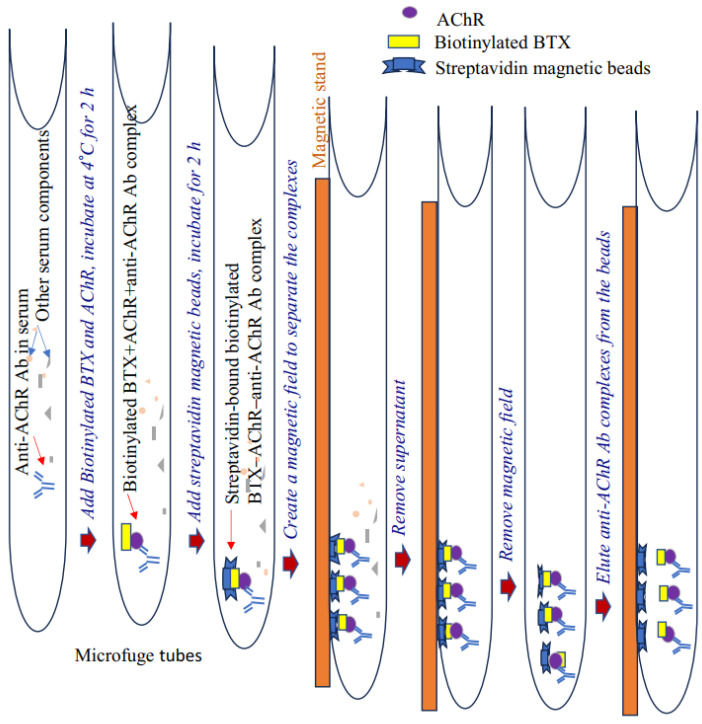
Schematic of a nonradioactive assay for detecting anti-AChR antibodies. Ab, antibodies; AChR, acetylcholine receptor.

Our assay accurately detects and measures relative serum levels of AChR autoantibodies in the serum of EAMG mice. The antibody titer was also validated through conventional ELISA [12]. This nonradioactive method appears more specific and efficient than conventional ELISA to detect high-affinity AChR-specific antibodies in samples. A schematic of the assay steps is presented in Figure 1.

### 2.3. Assay Steps (Time Required: Approximately 2 to 4 h)

#### 2.3.1. Immunoprecipitation

##### Materials

- PBS;
- PBS–Triton X-100 buffer (0.01% Triton X-100 in 1× PBS = 40 μL of Triton X-100 in 400 mL of 1× PBS);
- Affinity-purified Torpedo/mouse AChR—in-house purified (11);
- Alpha-Bungarotoxin Conjugates (#B1196; Invitrogen, MA, USA);
- Phenylmethylsulfonyl fluoride (Sigma, MO, USA), 100 mM in EtOH stock solution;
- Protease inhibitor cocktail (Sigma);
- Magnetic streptavidin beads (#5917; Cell Signaling Technology, MA, USA);
- Magnetic stand/rack (Invitrogen/StemCell Technologies, MA, USA).

##### Procedure

1. Add 1 mL of 0.01% PBS–Triton X-100 buffer to prelabeled 1.5 mL microfuge tubes placed on ice. Triton X-100 (detergent) is necessary in order to separate magnetic beads on the tube surface efficiently.
2. Add 2 μL of purified Torpedo or mouse AChR (concentration: 1 μg/mL), 2 μL of biotinylated BTX, and 3 μL of serum (from CFA- or CFA/AChR-immunized mice) to each tube. In addition, add 10 μL of phenylmethylsulfonyl fluoride (100 mM) and 10 μL of protease inhibitor cocktail. Incubate the tube on a rocker in a cold room for 2 h for immunoprecipitation.
3. Prerinse the streptavidin magnetic beads with 300 µL of 0.01% PBS–Triton X-100 buffer. Preclearing is unnecessary for some beads (e.g., Dynabeads [Pierce]). Gently pulse-vortex the beads and add 3 μL of bead suspension into each tube. Wrap the tube lid with parafilm (to prevent leakage) and incubate for 2 h to overnight at 4 °C on a rotator.
4. Briefly centrifuge the sample at a low speed (500 g) to move any solution from the lid into the microfuge tube. Place each tube on a magnetic stand/rack (Invitrogen/ThermoScientific) and incubate for 5 min to separate the beads from the extract.
5. Carefully and gently aspirate (manually or with a vacuum aspirator) to remove the supernatant without disturbing the pellet in the microfuge tube.
6. Add 1 mL of 0.01% PBS–Triton X-100 (wash solution) to each tube. Remove it from the magnetic stand, tap to resuspend, and return to the stand. Incubate for 5 min and then discard the supernatant, leaving the beads in the tube.
7. Repeat step 6.
8. Add 15 μL of PBS to each tube. Resuspend the beads in the buffer by tapping the tube.

#### 2.3.2. Immunoblotting (Any Standard Method; Time Required: Approximately 6–8 h)

##### Materials

- Nondenaturing 4× Laemmli sample buffer (#1610747; Biorad, CA, USA);
- Mini-PROTEAN TGX Precast Gels (4–20%; #4561095; Biorad);
- Rainbow marker (Invitrogen, MA, USA);
- Tris-buffered saline (TBS50 mM Tris–HCl and 150 mM NaCl; pH: 7.5);
- Polyvinylidene fluoride membrane (Biorad);
- Tris–glycine transfer buffer (Biorad);
- Methanol (Sigma);
- Bovine serum albumin (Sigma);
- Secondary antibodies: sheep antimouse IgG–horseradish peroxidase antibody (HRP; #ab6808; abcam, MA, USA) or goat antimouse IgG2b (#M32407; Caltag Laboratories, Burlingame, CA, USA);
- Enhanced chemiluminescence (#32109; Pierce Biotechnology, MA, USA) reagent;
- Amersham Imager 680 (Amersham, NJ, USA).

##### Procedure

1. Add 4 μL of 4× nonreducing sample loading buffer to each sample tube and tap to mix.
2. Incubate each sample in a heating block (at 95 °C) for 5 min. Magnetically separate the beads and collect the supernatant containing the AChR-antibody-containing immune complex (optional). The supernatant can be stored at −80 °C for up to a month for immunoblot analysis.
3. Load the samples together with beads (at room temperature) onto a 4–20% precast gel. In addition, load a rainbow marker (7–10 μL) into a gel well.
4. Run the gel in TBS running buffer at 125 V for 1 h.
5. To avoid a nonspecific background on the membrane, carefully slice off the bead-containing wells from the gel using a razor blade. Gently remove any remaining beads on the gel with water from a squirt bottle.
6. Follow any standard immunoblot transfer protocol to transfer protein from the gel to a polyvinylidene fluoride (PVDF) membrane.
7. After transfer, wash the wet PVDF membrane with distilled water 6 to 7 times to remove the methanol used in the transfer buffer. Label the front/back side of the membrane.
8. Rinse the membrane in TBS once and replace TBS with approximately 25 mL of blocking buffer (1% bovine serum albumin in 0.5% TBS-Tween 20). Incubate the membrane for 30 min on a rocker.
9. Wash the membrane and incubate with secondary antibodies (sheep antimouse IgG-HRP; dilution: 1:5000 [1 μL of secondary antibody in 5 mL of blocking buffer]) on a rocker for 1 h at room temperature.

The membrane can also be probed with an HRP-conjugated goat antimouse IgG2b (Santa Cruz Biotechnology [Santa Cruz, CA, USA] and Caltag Laboratories; dilution: 1:5000 [or an optimal dilution depending on the antibody titer in a lot]) in TBS-Tween 20. Anti-biotin-HRP antibody is not used to prevent partial dissociation of biotin from the beads during the elution step.

**Figure 2:**
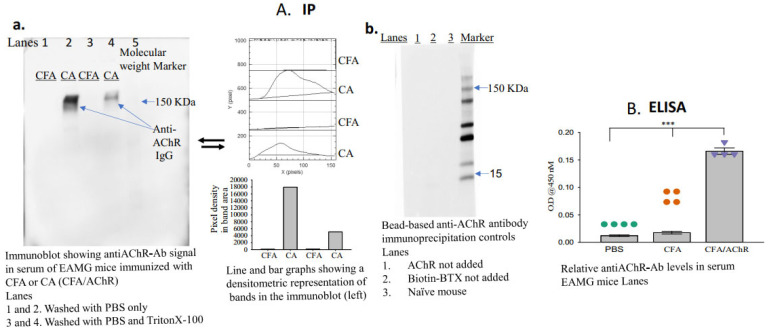
Serum levels of anti-AChR antibodies in mice immunized with CFA or CFA/AChR. (**A**) Serum levels of pathogenic anti-AChR antibodies were measured using the newly developed immunoprecipitation–immunoblot assay. The image on the left of A (**a**) is a representative blot from 3 experimental sets; samples in each lane were pooled from each group (*n* = 4). Densitometric analyses of the bands are also presented. The image on the right of A (**b**) represents an immunoblot using serum samples from naïve mice and EAMG mice (pooled from three samples per group). No anti-AChR antibody band was observed in the absence of AChR or biotinylated BTX in the assay. (**B**) Plot showing relative levels of anti-AChR antibodies; the levels were measured through enzyme-linked immunosorbent assay. The vertical bar represents the mean ± standard error of the mean (SEM), and calculated *p*-values denote significance at *p* < 0.001 (***). *n* = 4, represented by colored dots in each group. Each experiment was performed in three replicates. AChR, acetylcholine receptor; BTX, α-bungarotoxin; CFA, complete Freund’s adjuvant; Ig, immunoglobulin.

10. Wash the blot thrice for 5 min with 0.5% TBS-Tween 20 buffer (approximately 15 mL).
11. Apply the enhanced chemiluminescence reagent, image the blot (using a Gel Imager, Amersham imager 680, GE Healthcare Life Sciences, Little Chalfont, Buckinghamshire, UK), and measure the density of each band (ImageJ.net 1.53F, National Institutes of Health, Bethesda, MD, USA) representing the titer of anti-AChR autoantibodies in each sample (Figure 2A(a,b)).

The levels of anti-AChR antibodies in the EAMG mice can be verified through ELISA following our previous protocol [11]. Briefly, a pre-titrated serum dilution is reacted with affinity-purified mouse muscle AChR coated on a plate. HRP-conjugated rat antimouse IgG2b is used as a secondary antibody (Caltag, CA, USA, and BD Biosciences, Milpitas, CA, USA), and 2′-azino-bis (3-ethylbenzthiazoline-6-sulfonic acid) or 3,3′,5,5′-tetramethylbenzidine (ThermoFisher, Waltham, MA, USA) as substrates to measure the colorimetric output as a measure of autoantibody titer.

### 2.4. Statistical Analysis

Pooled samples from at least three replicates per group can be used for multiple groups. Groups are compared, and data are evaluated using the one-way analysis of variance test, followed by the Sidak or Tukey post hoc test. Statistical significance at *p* < 0.05 and *p* < 0.001 is used. All experiments can be performed in triplicate.

## 3. Results

A schematic of the newly developed assay is presented in Figure 1. The groups (*n* = 4, per group) received either AChR emulsified in CFA and PBS (CA) or CFA emulsified in PBS (disease control). Blood samples were collected two weeks after the second booster immunization to assess the presence of anti-AChR autoantibodies. The bead-based assay successfully detected anti-AChR Ab in the serum of TAChR-immunized EAMG mice but, as expected, not in CFA-immunized mice (Figure 2A(a)). To further test the specificity, the assay was performed using serum samples from groups of naïve mice or EAMG mice without the addition of AChR or biotinylated BTX in the assay. The results revealed no AChR-antibody-specific bands in the immunoblot in any of the lanes (Figure 2A(b)). Triton at a high concentration removes the binding of the AChR-antibody to the bead complex, so the band in the immunoblot is lighter than the one for PBS. However, a low concentration of Triton facilitates the separation of the beads from the nonspecific binding to the other serum components without affecting the band density. Therefore, we started using low-concentration triton for the assay, as described in the protocol.

## 4. Discussion

Anti-AChR antibody is currently the only established biomarker of AChR-seropositive MG. Because of the absence of any other reliable biomarker, the only option is to compare the autoantibody levels for disease diagnosis, severity assessment, and treatment response evaluation, both in clinical settings and preclinical laboratories. This underscores the importance of the accurate and consistent measurement of autoantibodies in MG.

Studies assessing AChR-specific autoantibodies have compared ELISA, CBA, and RIPA in terms of specificity and sensitivity [13,14,15]. All these classical diagnostic assays can differentiate patients with MG from those with similar clinical presentations but different disorders. However, these assays vary in terms of sensitivity, complexity, variability, and inconsistency. Owing to its relatively high (eight-fold increase) sensitivity, CBA is regarded as the first-line diagnostic test for MG; however, there have been concerns about the inability of CBA to determine the antibody titer as a non-cytometry assay. Furthermore, quantitative inconsistencies have been observed in follow-up assays in anecdotal reports. Thus, CBA serves as a second-line test when RIPA fails to detect autoantibodies in seronegative patients. Many other in-house developed assays are also not consistent. A concrete decision remains to be made by the MG consortia regarding the issuance of guidelines on a standard assay for measuring autoantibodies for disease diagnosis, monitoring, and treatment evaluation in patients with MG and preclinical MG models.

Our bead-based non-radioactive assay presents a significant advancement in AChR Ab screening. By avoiding the use of costly and hazardous radioactive reagents, the culture of transient or stably transfected cells, the risk of saturation reaction and non-specific reaction with plate-coated antigen, and the handling of sophisticated instruments, we have developed a simple, inexpensive, and nonhazardous assay. The data are visible and reliable, and the results are reproducible, making this assay a dependable, cost-effective alternative to conventional AChR Ab screening assays.

Our results present a proof-of-concept for the potential use of this newly developed immunoprecipitating–immunoblotting method for detecting and quantifying anti-AChR antibodies in serum samples from EAMG models and patients with MG. The results obtained using our assay are presented in Figure 2. We validated the binding of both anti-AChR antibodies and biotinylated BTX with AChR during the immunoprecipitation reaction. AChR has multiple binding sites, which explains the observed noninterference during the simultaneous binding of anti-AChR antibody and BTX [16].

## 5. Conclusions

To date, the anti-AChR antibody is regarded as the hallmark of MG diagnosis and treatment response evaluation, despite the debate on whether autoantibodies can be regarded as a reliable and sole biomarker of MG; this is due to the seronegativity of any autoantibody in at least 10% of patients with clinical evidence of MG [8]. The lack of autoantibodies is often attributed to the insufficient sensitivity of antibody detection methods, low-affinity autoantibodies, or the presence of antibodies against an unknown target antigen. Further, positive or negative outcomes of some MG treatments are reported to not always correlate with changes in the autoantibody titer [17]. Thus, in addition to developing an effective, highly sensitive, specific, and reproducible autoantibody assay, there is also a critical need to find unique biomarkers for diagnosis, proper management, and therapy evaluation.

In sum, we described an alternative and useful assay for detecting and measuring anti-AChR autoantibodies in the preclinical MG model. The further development and optimization of this assay (ongoing in our laboratory) may facilitate its application in the diagnosis of MG and other antibody-mediated diseases. Additionally, we are testing fluorochrome-conjugated secondary antibodies for the direct in-gel quantitation of anti-AChR autoantibodies.

## Data Availability

Data will be made available on request to the corresponding author.
